# Examining the militarised hierarchy of Sierra Leone’s Ebola response and implications for decision making during public health emergencies

**DOI:** 10.1186/s12992-023-00995-w

**Published:** 2023-11-22

**Authors:** Samuel T. Boland, Dina Balabanova, Susannah Mayhew

**Affiliations:** 1 Centre for Universal Health, Chatham House, 10 St James’s Square, London, SW1Y 4LE UK; 2 Department of Global Health & Development, London School of Hygiene & Tropical Medicine, 15-17 Tavistock Square, London, WC1H 9SH UK

**Keywords:** Sierra Leone, Ebola, Securitisation, Militarisation, Humanitarian intervention, Public health emergencies, Localisation

## Abstract

**Background:**

In September, 2014, Médecins Sans Frontières (MSF) called for militarised assistance in response to the rapidly escalating West Africa Ebola Epidemic. Soon after, the United Kingdom deployed its military to Sierra Leone, which (among other contributions) helped to support the establishment of novel and military-led Ebola Virus Disease (Ebola) response centres throughout the country. To examine these civil-military structures and their effects, 110 semi-structured interviews with civilian and military Ebola Response Workers (ERWs) were conducted and analysed using neo-Durkheimian theory.

**Results:**

The hierarchical Ebola response centres were found to be spaces of ‘conflict attenuation’ for their use of ‘rule-bound niches’, ‘neutral zones’, ‘co-dependence’, and ‘hybridity’, thereby not only easing civil-military relationships (CMRel), but also increasing the efficiency of their application to Ebola response interventions. Furthermore, the hierarchical response centres were also found to be inclusive spaces that further increased efficiency through the decentralisation and localisation of these interventions and daily decision making, albeit for mostly privileged groups and in limited ways.

**Conclusions:**

This demonstrates how hierarchy and localisation can (and perhaps should) go hand-in-hand during future public health emergency responses as a strategy for more robustly including typically marginalised local actors, while also improving necessary efficiency—in other words, an ‘inclusive hierarchical coordination’ that is both operationally viable and an ethical imperative.

## Background

By the late summer of 2014, the 2013–2016 West Africa Ebola Epidemic in Sierra Leone was overwhelming health systems. Consequently, in September, 2014, Médecins Sans Frontières (MSF) called for a militarised intervention in response to the escalating crisis [1]. Shortly thereafter, the United Kingdom (UK) government (HMG) announced Operation Gritrock, a bespoke military mission to support Sierra Leone’s Ebola Virus Disease (Ebola) response across a number of domains alongside the Republic of Sierra Leone Armed Forces (RSLAF). ‘Classical response actors’—defined here as civilian United Nations (UN); international and national non-governmental organisations ((I)NGOs); and national health actors—were thereafter placed directly alongside (and often under the direction of) British and Sierra Leonean military personnel in the daily management and operation of Ebola response activities within the militarised hierarchy of the National and District Ebola Response Centres (the NERC and DERCs, respectively).

Importantly, structural harms resulting from the deployment of militaries and classical response actors to Sierra Leone’s Ebola response also included the exclusion of many Ebola-affected communities from the response itself [2–4]. Local groups were substantively involved in responding to the Ebola outbreak within their communities [5], especially in the outbreak’s earlier days before the significant influx of military and classical response actors in the autumn of 2014. These community-level contributions are often un(der)recognised, but were significant in their nature and, arguably, effect [3, 6–9]. However, rather than systematically folding these local groups and their capacities into the formal civil-military response being organised by the Sierra Leonean government (GoSL) and the international community, these groups were usually excluded from it: the NERC and DERCs were certainly inter-agency spaces, but they were not always fully democratic ones, as routine participation was generally limited to classical response actors and militaries; doors were usually locked for meetings; and compound gates were often guarded by military personnel. Operational efficiency, after all, was felt by both classical response actors and the involved militaries to benefit from a hierarchical and top-down response. Ultimately, through the militarised Ebola response, the marginalised status of these local communities was (re)relegated to the subordinate. Furthermore, in reproducing these structural harms, the Ebola response was less effective for it, as robust community engagement is often cited as one of the most important factors in successfully responding to an epidemic of this nature [10].

Focusing on the use of hierarchy, this article considers the structure and operation of the NERC and DERCs with a view to identifying mechanisms through which the harms resulting from the militarised response were mitigated and partly undone.

## Framework

**Fig. 1:**
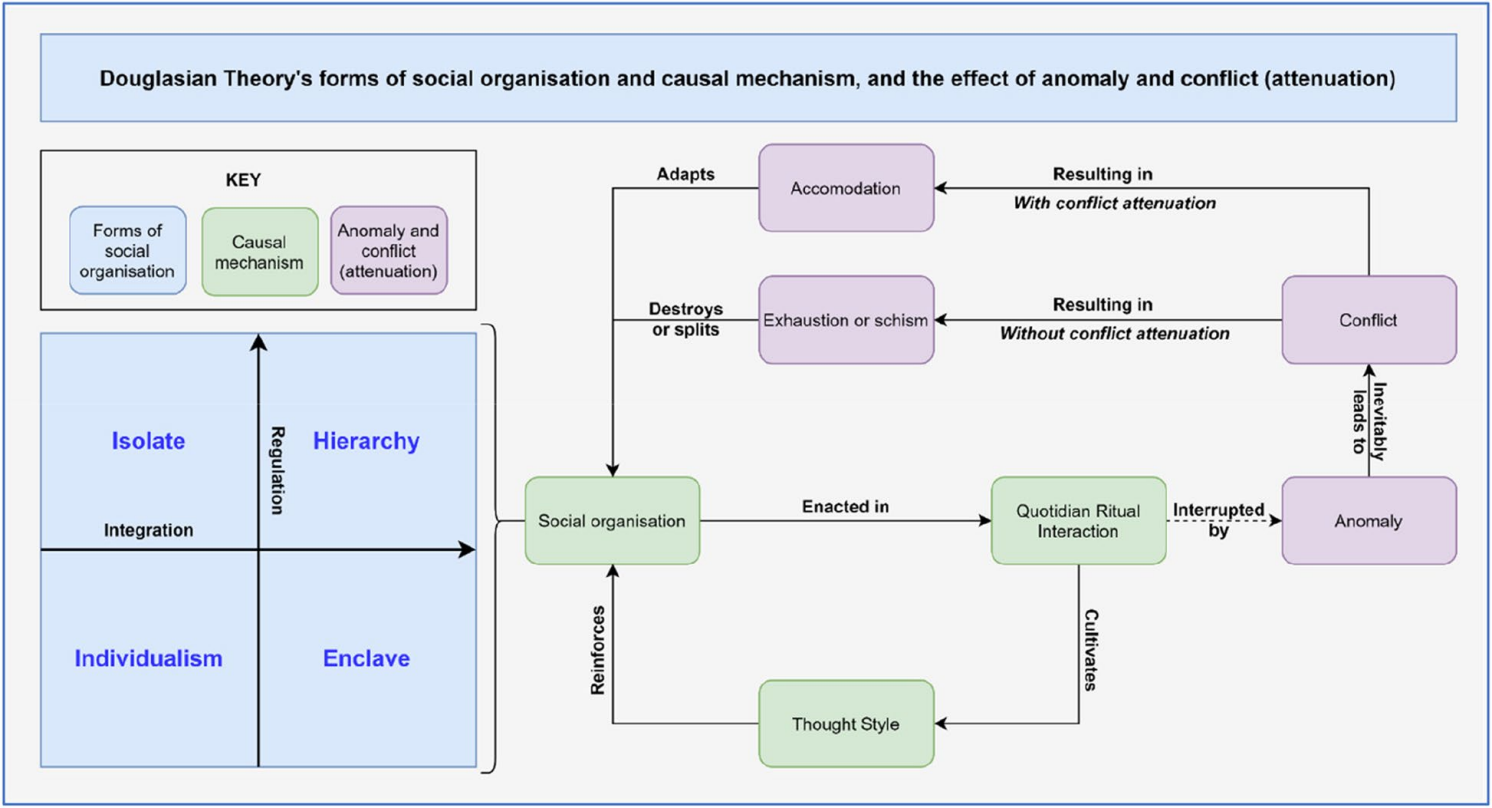
Douglasian social organisation; causal mechanism; and conflict (attenuation) (Source: author)

This article draws on the neo-Durkheimian theories of Mary Douglas (hereafter referred to as Douglasian Theory), which was chosen for its focus on understanding the way conflict inevitably arises between different groups of actors, as well as the ways this conflict can then be moderated. Douglasian Theory posits that there are four elementary forms of ‘social organisation’ distinguished by their varying degrees of social regulation and social integration (blue, Fig. 1). However, the forms are not mutually exclusive. In fact, many real-world social organisations exist as a blend of multiple forms representing an often lengthy and complex process of mutual accommodation [11]. Importantly, the four elementary forms of social organisation can describe not only a specific group’s internal organisation, but also the external spaces in which interactions between different groups occur and relationships manifest [5].

According to Douglasian Theory, a given form of social organisation is enacted in the mechanism of quotidian ritual interaction (that is, daily routine and interaction), which cultivates a ‘thought style’ [5]. The thought style, in turn, reinforces the social organisation that produced it, which is the final step in Douglasian Theory’s causal mechanism (green, Fig. 1).

In its extreme form, this can actually be a disorganising rather than reinforcing process, where the social organisation fails to accommodate internal and external pressures or ‘anomaly’, leading to exhaustion or schism [5]. Douglasian Theory posits that conflict within and between groups is inevitable for precisely this reason (purple, Fig. 1) [5].

However and crucially, the theory also identifies four specific mechanisms of hierarchical ‘conflict attenuation’ that can disrupt (or at least mitigate) this process, leading instead to the eventual accommodation of anomaly by enabling quotidian ritual interaction to continue in an adapted way (Table 1). Conflict attenuation can therefore also serve to improve deconfliction and facilitate cooperation and collaboration between different actors (even across the four forms of social organisation).

**Table 1:** The four mechanisms of hierarchical conflict attenuation

Conflict attenuating mechanism	Description	Relevance to this study (illustrative examples)
**‘Rule-bound niches’**	The permitted presence of another social organisation, provided it observes hierarchically defined boundaries and only operates within its authorised or sanctioned space	Different groups having different and delineated scopes of work/activities within the NERC and DERCs’ pillar system, such as the World Health Organisation (WHO) overseeing surveillance or an (I)NGO overseeing the alerts desk, such that no one group conflicted with or overrode another
**‘Neutral zones’**	Agreed spaces in which different social organisations can co-exist without threatening the existence of another	The NERC and DERC meeting spaces where different groups could come together to discuss daily activities and resolve challenges collectively
**‘Co-dependence’**	When different social organisations are interdependent and mutually co-constitutive	Civilian and military Ebola Response Workers (ERWs) that had to work together with the shared objective of containing the Ebola outbreak
**‘Hybridity’**	Where the constitution of a given social organisation imbricates with another	Civilian ERWs becoming more hierarchical and military ERWs becoming less so, making them more like the other

While each form of social organisation is capable of conflict attenuation, Douglasian Theory is plain about hierarchy’s unique ability to accommodate other forms of social organisation using these four mechanisms of conflict attenuation [5].

In this article, Douglasian Theory will be utilised to examine the way Sierra Leone’s hierarchical Ebola response centres were conflict attenuating spaces that generally led to accommodation (rather than exhaustion or schism) between different organisations. It will ultimately show how the use of hierarchical conflict attenuation can be used to concurrently achieve operational efficacy and efficiency alongside—indeed, through—the robust inclusion of typically excluded and marginalised groups.

## Methods

**Fig. 2:**
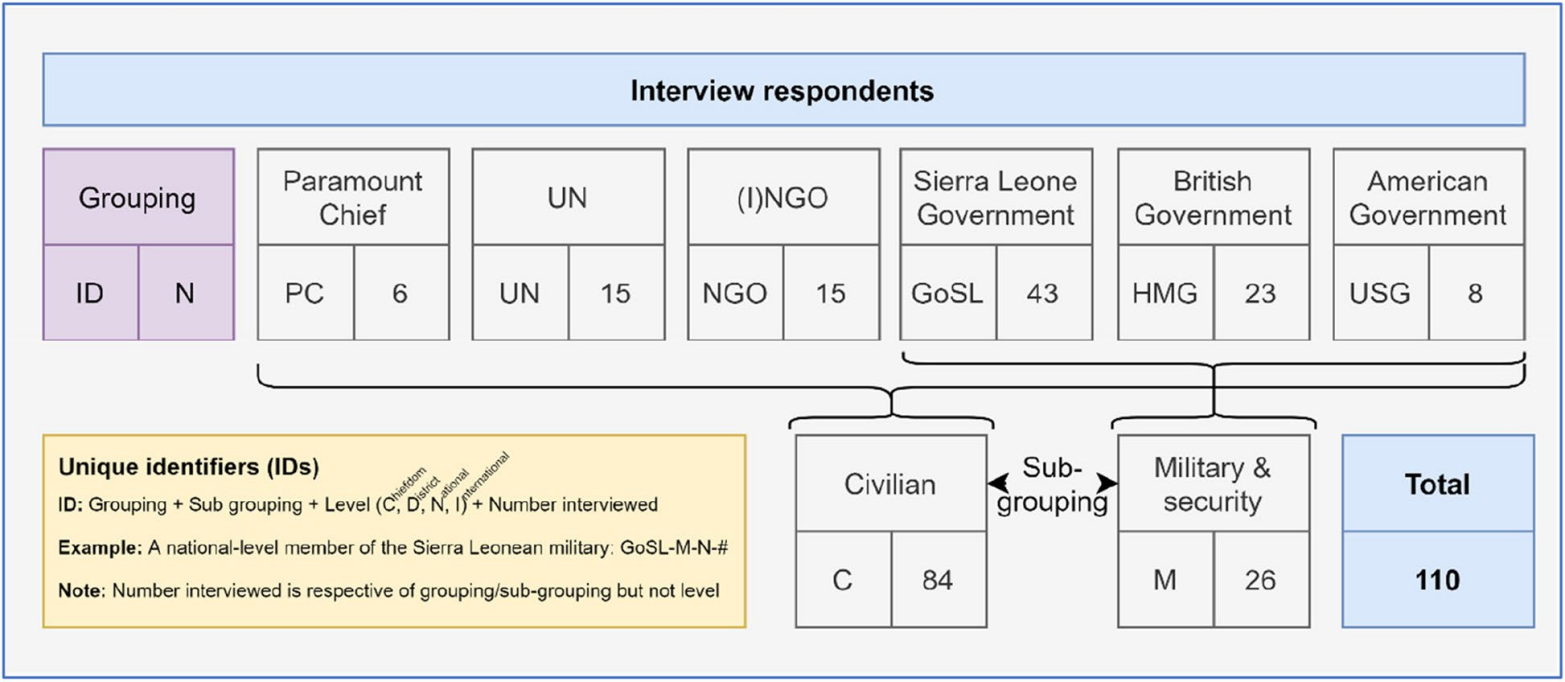
Interview respondents (Source: author)

This article relies on the analysis of 110 semi-structured qualitative interviews conducted by the lead author and researcher (STB, male) between 2017 and 2018 (Fig. 2) (research was conducted by STB as a doctoral candidate as supervised by DB and SM). Site selection included Western Area Urban District (i.e., Freetown) so as to collect national-level perspectives; and Port Loko and Kambia districts, so as to collect (sub-)district-level perspectives. These districts were chosen due to STB’s extensive experience working in the districts during the 2013–2016 West Africa Ebola Epidemic, which helped facilitate access during data collection.

Subject selection was purposefully broad so as to reach saturation. It included anyone who was involved in the Ebola response at these research sites who was either affiliated with the NERC and DERCs or with the activities being coordinated within. A specific focus was given to maximising the diversity of respondents’ organisations, agencies, or departments (*n* = 41). Respondents were approached face-to-face where possible, and telephone or email where not. As detailed in Fig. 2, each respondent was assigned a unique identifier (ID). A number of respondents—especially initial respondents, from whom subsequent respondents were identified in a general snowballing method—were known to STB from their prior work. This facilitated access and openness, but may also introduce biases which are reflected on later in this article. The study included a total of 110 respondents (none refused to participate or dropped out of the study at a later time).

An interview guide was developed and utilised for all interviews, which were semi-structured and open-ended in nature. The guide was developed in a primarily inductive manner, with themes incorporated iteratively as they arose through the interview process. Interviews—which were approximately an hour long and recorded—were conducted at the place identified by the respondent as most convenient and comfortable, except where conducted remotely by telephone due to being overseas (the latter was particularly relevant for non-Sierra Leonean respondents who had since left the country). No other persons were present beside the respondent and STB. To assist with researcher reflexivity, notes were taken during interviews, and memos written after each. Interviews were transcribed by STB (these were not returned to participants for comment or correction except where the audio was unclear).

Initial organisation of the data drew on framework analysis: familiarisation was accomplished through the central role of STB in all aspects of this study; data was inductively coded in NVivo; nodes were then reviewed, aggregated, and disaggregated where appropriate; and data were then charted and mapped for patterns within and between respondent groupings. As previously described, Douglasian Theory was then applied to the data in order to interpret the findings.

Ethics approval was granted by both the London School of Hygiene & Tropical Medicine (LSHTM) Research Ethics Committee (reference #14424) and the Sierra Leone Ministry of Health and Sanitation (MoHS) Office of the Ethics and Scientific Review Committee (no reference number provided; approved August 28th, 2017). All research was conducted according to accepted norms for ethical research.

## Results

The findings fall into the examination of three main areas, presented in separate sections: how civil-military co-dependence was developed; how the other forms of conflict attenuation were utilised; and how these same tools were used to enable the Ebola response to be scaled to more local actors. Note, when ‘militaries’, ‘military actors’, or ‘military respondents’ is used as plural, this is intended to be understood as both the British and Sierra Leonean militaries taken together—where a specific military is being referred to, this is indicated.

### Developing civil-military co-dependence

This section first examines how classical response actors are frequently characterised by respondents as having various weaknesses that were deemed by some as detrimental to the Ebola response. Thereafter, how military actors nevertheless took proactive steps to incorporate classical response actors is examined, including through the implementation of oversight and accountability mechanisms. The section ends by examining how, taken together, this represents an important co-dependence that was developed between classical response and military actors within (and as facilitated by) the hierarchical NERC and DERCs.

#### Perceived classical response actor weaknesses

Before the intervention of RSLAF and the British Armed Forces (specifically in their support to the overhaul of national and sub-national Ebola coordination centres), “*it was complete smoke and mirrors”,* recalled an (I)NGO respondent (NGO-C-N-10). Indeed, respondents who were present in the Ebola response’s early days consistently recalled a fraught sense of lethargy, incoherence, and disorganisation in the MoHS and WHO-led Ebola Operation Centre (EOC) responsible for coordinating the country’s national response. District-level respondents often noted the same concerns in the District Medical Officer (DMO)-led District Health Management Teams (DHMTs) organising the country’s various district responses. According to an (I)NGO respondent (implicitly referencing the forthcoming change in national and district coordination):

*We went from a world where the EOC meeting would happen in the DMOs office with the WHO sitting there scratching their heads, and the burial team lead saying ‘we buried eight people today’—and I’m not joking—everybody giving* [them] *a round of applause, and then everyone moving onto the next subject. And I would say, hold on, hold on, but how many bodies were reported? And they’d say, ‘oh we don’t know’* (NGO-C-N-7).

To this respondent, the “*completely inadequate*” lack of accountability (in this case around dead body management) was a serious concern (NGO-C-N-7): reporting on the number of people successfully buried tells one very little about the success of the burial system in place, unless one knows and includes the relevant denominator—which, with bodies frequently lying to rot on the streets at this time, was unknown but plausibly quite high (NGO-C-N14, GoSL-C-N-24, HMG-C-I-5). This perceived disorganisation was congruous with the perspective of a GoSL civilian respondent, who—despite working for the MoHS—suggested that

*…when you look at the setup of our* [state] *ministries, in terms of the way operational activities are taken, you see a lot of delays. You see a lot of lethargy. People don’t meet timelines* (GoSL-C-N-20).

This important difference between classical response actors and military actors was illustrated by a senior (and ex-military) HMG civilian respondent who theorised about the root causes of the differences:

*The truth is, different kinds of people go into different kinds of professions. They have different ways of operating, and they have a different understanding of how the decision can and should be made… If you have a public health person, you can spend a lot of time discussing things, and they want everybody to have their say and to come to a joint consensus. That takes an extremely long time, and usually none of them are prepared to take the ultimate responsibility of making a decision and getting on with it. Military people are trained in a very simple way… The people in the military are trained to take responsibility, and then to act on it. That makes a big difference* (HMG-C-I-4).

In other words, to this respondent, the less hierarchical approach that generally characterises classical response actors represented a significant difference when compared with a more militarised one (a difference that in their mind was something to criticise, in that they considered the classical response actor approach to be relatively slow, cumbersome, and ineffectual). In the Sierra Leone context specifically, this perspective was widely shared across all respondent groupings (albeit often with less forceful disapproval). Classical response actors were frequently characterised in the data as being less efficient and less disciplined (particularly with regards to time management) than their military colleagues.

Accordingly, while respondents were not specifically asked to comment on what they perceived the weaknesses of classical response actors to be, relevant insights nevertheless emerged during some interviews. For example, many respondents noted issues such as inadequate efficiency, time management, and focus (*n* = 37), which was the second most common criticism after a lack of preparedness (*n* = 43). There was little differentiation between respondents’ grouping or level. Along with insufficient capacity (*n* = 36), classical response actors were also frequently perceived to manifest weak coordination (*n* = 21). This was a particularly problematic gap, as in the summer and early autumn of 2014, an increasing number of classical response actors were planning and preparing to deploy in response to the escalating crisis.

Both military and classical response actor respondents pointed to the weaknesses they perceived in classical response actors, which were ultimately one of the stated impetuses for the NERC’s replacement of the EOC (as well as the associated removal of MoHS staff from direct leadership over the Ebola response).

#### The militaries’ proactive incorporation of classical response actors

However, despite these perceived key differences—importantly, ones that military respondents often criticised—classical response actors would be not only integral to daily Ebola response operations, but integral in a way that largely resulted from the planning of several key personnel (including military actors) at the national level.

An (I)NGO respondent (who was one of the key figures involved in developing the NERC and DERC system) recalled being in a meeting where the centres were being conceptualised. Along with representatives from the British Armed Forces and RSLAF, they were

*…drawing… things like roles and responsibilities… on whiteboards,* [and deciding] *where we would put different organisations… We drew out all the process maps, and then we got them printed on to big pieces of paper… We made sense of the chaos… We’d been plotting and scheming for 2 weeks, mapping and planning, getting all the resources in place, working it out with RSLAF…* [We] *had the Red Cross ready, we needed Concern Worldwide… we needed others ready… So, we told* [all these groups] *‘you report to the* [new] *command centre from tomorrow morning’, and they just came!* (NGO-C-N-7).

This suggests that the small civil-military team that set up the NERC and DERCs designed roles for and then delegated responsibilities to classical response actors. Indeed, the intervention of the militaries and the creation of the NERC and DERCs had the specific objective of facilitating the deployment of classical response actors to the Ebola response. This is because military actors generally understood that classical response actors were integral to the process and ability of getting large-scale response activities up and running in a short period of time (a capacity that the militaries did not have). A national-level British Armed Forces respondent recalled:

*That is what the plan was. The backstopping* [of the] *international community, to say, ‘you can all come and help, all you humanitarians, come and do your job…. And the point is, it was 200 million* [Great British] *Pounds that guaranteed the international and NGO* [presence]*. So, when you weigh those* [financial costs]*, DfID obviously said ‘it’s worth it!’ Otherwise,* [the UK is] *not going to get these other* [non-military] *people. And the military can’t fill all of these other roles, or won’t. They won’t run six hospitals, they won’t do coordination with social mobilisation, they won’t do all the contact tracing, you know, we won’t do that* (HMG-M-N-5).

Therefore—despite their perceived weaknesses and the removal of MoHS and WHO leadership over the Ebola response—the default position of the response’s key military decision makers was that classical response actors were an integral and complementary part of the NERC and DERCs’ civil-military constitution.

Importantly, the militaries’ desire to proactively include classical response actors was not limited to a small number of key military decision makers at the country’s national level. Indeed, military respondents across the research sites frequently expressed a degree of humility regarding their lack of relevant technical and medical expertise in response to a kind of emergency (i.e., a public health one) that they were unaccustomed to. For example, according to one British Armed Forces respondent, some classical response actors spent

*…a lot of time trying to prove to us that they were the experts in what they were doing and that we should all bugger off and leave them alone. But actually, that was never questioned. We were never pretending that we were better than anyone else. We were just supposed to be there to support it happening, and to try and make it happen as well as* [the classical response actors] *could do* (HMG-M-D-8).

Accordingly, most military respondents at all levels saw their primary strength as “*the operationalisation of…* [classical response actors’] *nebulous ideas into day-to-day actions*” (USG-M-I-1), rather than the performance of those actions themselves. In other words, a British Armed Forces respondent stated that instead of taking over work from classical response actors, “*the military’s really added benefit was to stop* [them from] *navel gazing about how to respond and just to get on and respond*” (HMG-M-N-2).

In short, classical response actors were recognised by most military actors for not just their capacity but also their competencies and were thus purposefully assembled and incorporated into the Ebola response’s new militarised coordination centres.

#### The introduction of robust oversight and accountability mechanisms

The military respondents’ comments (above) show a degree of understanding that the military were not going to ‘run the show’, but rather, that they needed to help create a structure and an enabling environment into which classical response actors could arrive and perform Ebola response activities. This structure was felt to require strong accountability and oversight mechanisms in order to mitigate the previously described perceived weaknesses of classical response actors. In doing so, it was thought that classical response actors’ valuable contributions could be better realised, without risking the disorganisation and inefficiency that most respondents felt had characterised the EOC and DHMTs.

Said a British Armed Forces respondent:

*There’s a lot of people out there doing a lot of good things and we just* [have] *to make sure it’s all going in the right direction and to keep the momentum going… It’s almost like having a sweeping action, just sweeping behind everybody. Making sure that everyone is keeping the same direction… Somebody has got to be making sure that it’s all going down the single lane, to the single point* (HMG-M-D-4)

Accordingly—so as to align efforts in “*the single lane*” and focus on “*the single point*” objective of containing the Ebola outbreak (HMG-M-D-4)—in replacing the EOC and DHMTs with the military-led NERC and DERCs (respectively), the militaries *“came in and created a kind of rules-based system*” (NGO-C-N-10), in which “*the processes and the systems…* [and] *a series of SOPs* [standard operating procedures]” (NGO-C-N-7) for daily operations were established (in Douglasian terms, SOPs could be thought of as akin to ritual ordering). This, in turn, created a set of expectations (i.e., they identified the Ebola response’s various denominators), against which classical response actors’ day-to-day interventions were publicly measured at the NERC and DERCs’ morning and evening briefings.

A mechanism for “*ruthless accountability*” (HMG-C-D-6), suggested an HMG civilian respondent, was therefore established within the NERC and DERCs, which helped to ensure that the centres’ processes, systems, and SOPs were followed by the (increasingly various) actors operating within them, this fairly sudden shift caused some challenges for classical response actors). This discipline was taken especially seriously by the NERC and DERCs’ military actors. One, a senior RSLAF respondent, considered it a life-or-death matter comparable to battlefield orderliness:

*You have to be really disciplined* [when responding to an Ebola outbreak]*. Because if you rush the process, then you might miss some of the points, and you will be infected, and you will die. In the military, if you ask me to strip and assemble a weapon, I know what comes out first, and I know what comes out second, and I know what comes out last. And I know what goes in again first when I’m assembling* [it]*… You do it dogmatically, so you make no mistakes. Because if you make mistakes in placing the wrong part in the wrong position with your weapon, then you are a dead man…* [Therefore, in Sierra Leone] *the military was able to follow procedures dogmatically…* [In the NERC and DERCs,] *we… brought that discipline to the civilian workers…* [and] *reduced the number of deaths… It’s like a ritual, that’s the right word. Like a ritual* (GoSL-M-N-11).

“*To defeat Ebola*”, the RSLAF respondent continued and summed up, “*was just simple discipline*” (GoSL-M-N-11), acculturated through dogmatic and ritualised procedure, which in turn, cultivated a hierarchically ordered thought style.

Importantly and accordingly, though, the DERCs’ civil-military Command Team did not enforce this discipline within the NERC and DERCs (as described later, they did not have any formal authority to do so). Rather, they put daily rituals in place that facilitated it. As recalled by an HMG civilian respondent (and Command Team member), without the NERC and DERCs,

…*you wouldn’t have had a morning* [or evening] *brief*[ing]… [and therefore] *you wouldn’t have had a sense of urgency, and a sense of accountability…* [The NERC and DERCs]*… put these elements together* (HMG-C-D-6).

In other words—through morning and evening briefings and other hierarchical processes, systems, and SOPs—it was felt that the NERC and DERCs provided the hierarchical structures within which this discipline could be cultivated and ritually acculturated amongst classical response actors.

For this, the NERC and DERCs were widely commended by respondents regardless of their grouping or level (re-noting, though, that with the exception of Paramount Chiefs, subject selection criteria as previously described meant the majority of respondents were associated with one of these centres and usually compensated for their work within). For example, a UN respondent recalled how it was “*refreshing to have predictability and reliability and accountability*” within these centres (as they felt there had previously been very little) (UN-C-N-3). An (I)NGO respondent recalled how “*people really responded to the structure and discipline*” (NGO-C-N-7) that was imparted. One RSLAF respondent even recalled how the occasional nagging and cajoling by military actors that was required to keep things moving in the DERCs quickly became such a trope that, in their memory, classical response actors and military personnel would sometimes take a step back and “*crack funs* [*sic*] *and… just joke and laugh*” about the militarised oversight (GoSL-M-D-4).

#### Co-dependence by (civil-)military design

The militaries’ intervention was felt by many to be significant for the enabling environment it created in the NERC and DERCs, because within these civil-military centres, the diverse number of activities conducted by a range of actors could be better directed towards the shared objective of containing the outbreak. In other words, the British Armed Forces and RSLAF proactively built co-dependence into the very design of the NERC and DERCs, even though many military actors felt that classical response actors could be cumbersome and inefficient in their decision making processes as examined above. Having these hierarchical structures and co-dependent procedures in place, recalled a GoSL civilian respondent, helped to allow for

*…the NGOs, the* [I]*NGOs, the WHO, UNICEF,* [and other classical response actors to intervene]*…* [and allowed for] *all these organisational resources* [to be] *poured in. And they were swift to move, so that the response was a rapid response* [that could] *alleviate the situation and save lives* (GoSL-C-N-26).

Moreover, once the resources were “*poured in*” (GoSL-C-N-26), the same structures—in the words of a British Armed Forces respondent—

*…forced everybody to work together. Because we had to. And I think that in a lot of cases the civilian organisations recognised the ability of… that structure* [as one] *within which* [they could] *do their job* (HMG-M-D-4).

This was further echoed by an (I)NGO respondent, who recalled:

*Ebola is no friend of any of us. And this could never happen without the right knowledge and expertise. And so, you won’t be able to do these things without the UN agencies, nor should we. But the military were critical sitting at the table. We needed people who were ready to move and turn a policy into an implementation plan* (NGO-C-N-7).

Thus, within the hierarchical NERC and DERCs, the militaries were felt by both civilian and military respondents to provide the necessary oversight of a growing and increasingly complex, multifaceted, and multi-actor Ebola response, representing a significant degree of co-dependence between the involved military and classical response actors. Accordingly, as measured across all respondent groups, four of the most frequently cited positive attributes of military ERWs were the control (*n* = 42); discipline (*n* = 45); efficiency, time management, and focus (*n* = 47); and overall strength in coordination (*n* = 51) that they manifested within and acculturated throughout the NERC and DERCs. The centres’ other conflict attenuating mechanisms (to be subsequently examined) helped classical response actors to continue practicing their quotidian ritual interaction and thereby encouraged the accommodation of this anomaly and preservation of their social organisation.

### Other forms of hierarchical conflict attenuation in the NERC and DERCs

Focusing on the perspective of respondents situated within the NERC and DERCs, how each other conflict attenuating mechanism was employed within these centres is examined. This is done with a view to understanding how anomaly was eventually accommodated.

#### Rule-bound niches and neutral spaces

As previously described, during the development of the NERC and DERC structures, it was recognised that the Ebola response required not only the provision of medical care to infected patients, but numerous other interventions as well. A respondent involved in the design of these structures noted that

*…it was only when the tyres hit the road that we looked… and went okay, so, we need burials, we need surveillance, we don’t have anyone looking after quarantine [and] someone needs to be doing quarantine… Why don’t we have* [each] *as a specialist area?* (NGO-C-N-7).

Therein, it was decided that each intervention should be organised as a specialist area bounded by a specific and rule-bound scope of work. Accordingly, a bespoke structure—the pillar system—was created, in which each intervention formed an operationally distinct pillar within the wider system (e.g., surveillance, dead body management, and logistics).

A specific classical response actor was made primarily responsible for forming, managing, and operationalising each. Some—such as the case management and security pillars—were run by medical or military actors, and were therefore quite hierarchical in nature. Others—such as the social mobilisation and psychosocial pillars—were run by (I)NGOs focusing on community engagement and a degree of local ownership, and were therefore more horizontally organised and consensus-driven. The different approaches followed from the perceived need for clear and efficient procedures in some activities (such as those which required rapid intervention to prevent onward transmission from known cases), and the perceived need for a greater degree of exchange, debate, and conversation in others (such as those which focused on slower processes of community behaviour change). Taken together, the pillar system (which comprised the NERC and DERCs) served to organise the various Ebola response interventions being managed and performed by diverse actors on a day-to-day basis. Therefore, these centres embodied the use of rule-bound niches.

In Port Loko and Kambia districts, these pillars were organised underneath and coordinated by a civil-military Command Team which was comprised of six individuals: a military representative from the British Armed Forces and RSLAF; an HMG civilian from the Stabilisation Unit (SU); a Sierra Leonean District Coordinator (DC) (appointed by the President); the WHO Field Coordinator; and the DMO. The Command Team’s mandate was to help ensure that the various pillars’ work streams were effectively aligned towards the shared objective of containing the outbreak and also to up-report on daily activities, challenges, and the changing epidemiological situation to the NERC in Freetown.

The interactions that occurred between the Command Team and (rule-bound) pillar leads in the NERC and DERCs occurred within a neutral zone (defined by Douglasian Theory as a space “in which negotiations might be sustained, but where none of the forms has a power of absolute veto or insistence”) [5]. During daily interactions and morning and evening briefings: the day’s activities were reviewed; challenges discussed and possible resolutions offered; and the subsequent day’s activities were planned and coordinated. The Command Team chaired day-to-day interactions and the evening *tour-de-table* discussions, but as described, generally maintained an oversight and accountability role. While formally this process may have imbued the Command Team with a degree of power, it did not intervene in specific pillar activities which were considered the domain of classical response actors.

Accordingly and importantly, the Command Team did not have the formal authority to direct any particular organisation (e.g., the WHO), only to advocate for recommended changes (HMG-C-D-6). In other words—despite its moniker and in line with the NERC and DERCs’ constitution as neutral spaces—the Command Team role was neither to veto nor to insist. One HMG civilian respondent (and member of a Command Team) acknowledged this lack of direct control by enquiring:

*How do you promote accountability across* [an] *organisation that you have absolutely no formal control over, when you* [are] *just trying to build consensus?* (HMG-C-D-6).

In generic terms, the respondent did go on to describe how they felt the Command Team was able to facilitate this accountability and build consensus amongst classical response actors through aligning and focusing their work:

*It was a serious situation, and I think everyone felt it was a serious situation. And… there was this certain* [energised] *vibe that I don’t think* [the Command Team] *generated. I think it was self-generated amongst the people there. But we were able to corral it… and marshal it…* [and make sure] *that everyone was on the same page* (HMG-C-D-6).

More specifically—as recalled by another Command Team member from British Armed Forces—the Command Team

*…would lead…* [but] *try to do so in a way where everybody in the audience knew that they were playing a part, and that they* [were] *a part of the decision making process… and…* [then] *just bring… together every brilliant idea and put it into a plan* (HMG-M-D-4).

In doing so, the Command Team: convened classical response actors; helped to “*put* [the response’s] *elements together*” (HMG-C-D-6); and therein, helped “*turn* [their various] *polic*[ies] *into an implementation plan*” (NGO-C-N-7) that better aligned their collective efforts (each of which was being independently operationalised within a pillar as above, and therefore possibly at risk of dissonance without this kind of stewardship). As summarised by a GoSL civilian respondent: “*Let us not forget* [that the DERCs’] *military* [actors in the NERC and on the DERC Command Teams] *just helped us to organise and plan… with a kind of coherence*” (GoSL-C-N-17).

The extent of differentiation between roles and function was therefore significant, both between the rule-bound pillars themselves, and also between the Command Team and the pillars that they oversaw; the Command Team helped to facilitate others’ activities within their rule-bound niches and worked within the NERC and DERC’s neutral space to discuss and resolve problems that arose as well as to align interventions towards a common goal. This helped to ensure that classical response actors were able to continue practicing their interventions and, thus, continue manifesting their quotidian ritual interaction and reinforcing their social organisation.

#### Co-dependence, hybridity, and the coupling of shared interests

Crucially and further, by differentiating functions between classical response actors and the involved militaries through the pillar system in this way, the NERC and DERCs’ daily operation required co-dependence (which was examined at greater length in the previous section): while any given pillar represented a specific scope of work, taken together, they comprised the Ebola response. In other words, each pillar was a fundamental component of the whole, and all had to operate not only concurrently but also in concert for the Ebola response to manifest and function.

As this co-dependence demanded that diverse actors work together, a degree of hybridity (that is, a degree of “melding” or “blending” between different social organisations) was necessitated [5]. Douglasian Theory argues that hybridity demands compromise by interacting groups, in that they must incorporate (rather than confront) each other’s thought styles [5]. Accordingly, among a majority of both military and civilian respondents, there was an understanding that a fully militarised command and control (C2) (i.e., C2 in the way that a military might typically understand and apply it internally) was not always appropriate when engaging with classical response actors in a multi-agency and civil-military response.

Equally, however (and as previously described), it was also felt that classical response actors’ perceived disorganisation and inefficiencies were unsuitable in the response to a highly dynamic and life-threatening crisis. Therefore, instead of one approach fully dominating, over time, many of the different actors in the NERC and DERCs became more alike one another. That is to say, Militaries became less hierarchical in nature, and classical response actors became more so. This was captured by a GoSL respondent who argued that

*…mixing people* [in these centres] *broke down barriers by encouraging people to learn from each other… As soon as* [civilian and military personnel] *started working together, the civilians started appreciating the fact that the military did things rigorously and they very quickly picked that up. So,* [over time]*, if* [the Command Team] *said six o’clock, it was six o’clock,* [and the] *civilian staff were there. They were punctual, and they became just as organised and strategic as the military mind is. And at the same time, the military learnt to be more compassionate, learnt to be less rigid, learnt to debate things which they don’t generally do in the army* [*laughing*]*. And they learnt to work with local communities better…* [as] *they now underst*[ood] *that it is not always the case that you just give orders and then… things* [get] *done* (GoSL-C-N-17).

Indeed, the military analogue to the DERC—that is, a place from where localised interventions are operationalised—is called a Forward Operating Base (FOB). In a military FOB, a C2 approach (including the rule of law and following orders) is a non-negotiable *modus operandi*. Therefore, from the perspective of military actors involved in Sierra Leone’s Ebola response, the purposeful and proactive inclusion of classical response actors in the NERC and DERCs meant these centres were atypically consensus-driven, horizontal in organisation, democratic in function, and inclusive in nature (however, of note, the British Armed Forces was not deployed to Sierra Leone *tout corps*. Personnel—who were unarmed—included military medical staff, technical experts, engineers and logisticians, and administrators, for example. Operation Gritrock did not include combat troops, who may be more accustomed and adhere more strictly to military hierarchy. For example, one British Armed Forces medic (HMG-M-D-8) noted that, in their experience, medical hierarchy supersedes military hierarchy due to the technical nature of their expertise).

A British Armed Forces respondent echoed how the civil-military inter-agency collaboration required this softening of approach, saying

*…thankfully, we have politicians to balance the military alpha male with the political expediency, with the public opinion,* [and] *with the humanitarian workers. So,* [between] *all sides—you know, politics, military, lobbying, humanitarian—you… come up with a middle ground* (HMG-M-N-5).

In other words (and as discerned by Douglasian Theory), classical response actors and military actors operating in isolation were prone to the reinforcement of their own social organisation and thought style. Acting in concert, though, mitigated the degree to which this occurred within a given group, as extreme forms of social organisation were moderated through hybridity in a conflict attenuating way. By becoming more hierarchical, classical response actors were able to accommodate the anomaly presented by the unusually hierarchical and civil-military Ebola response. In becoming less so, military actors were able to accommodate the anomaly presented by the ways the response was atypically horizontal, consensus-driven, and inclusive of non-military actors. Hybridity thus helped more respectful relationships between involved actors to develop, wherein a degree of mutual learning enabled them to not only recognise the strengths and weaknesses of their different approaches, but to put this learning into practice within the NERC and DERCs.

According to Douglasian Theory, successful co-dependence and the kind of organisational hybridity seen in the NERC and DERCs is significantly aided by the coupling of shared interests [5]. In the Sierra Leone case, this was straightforward: differently organised actors not only worked alongside one another, but did so while sharing the unambiguous (and bounded) objective of containing the Ebola outbreak. As stated by an HMG civilian respondent:

*There was no military or political strategic imperative other than how do you help stop this potentially ravaging outbreak as quickly as possible. It was* [as] *simple as that* (HMG-C-N-14).

In line with Douglasian Theory, one GoSL civilian respondent felt that having a shared objective in this way was of central importance to actors coming together peacefully, noting how there was

*…a camaraderie which identified one enemy, Ebola. Ebola was an enemy of our country, and was killing our people. And recognising that it is us versus the virus, and* [that] *this is an existential threat, a do or die situation… That helped people to coalesce together* (GoSL-C-N-17).

Most respondents (regardless of their grouping or level) agreed that—despite any differences between them—civilian and military ERWs were joined by the primacy of this shared objective (though a number of primarily international-level respondents raised concerns about a possible ulterior motive of military actors, in that they plausibly expanded their role in responding to the Ebola outbreak in Sierra Leone. Meanwhile, a large number of both civilian and military respondents at all levels raised concerns about the organisational and financial security afforded classical response actors by the Ebola response, representing a possible conflict of interest). As the NERC and DERCs were the organising spaces in which the shared objective of containing the outbreak could be focused and realised, they were foundational to the successful development of its actors’ conflict attenuating co-dependence and hybridity.

Taken together, for their use of rule-bound niches, neutral zones, co-dependence, and hybridity, the NERC and DERCs can be understood as not merely hierarchical organisational structures, but as conflict attenuating ones. Clear, rules-based boundaries and procedures were established, which were manifested and negotiated within neutral spaces. This amounted to a necessary co-dependence, which in turn (and as further facilitated by the coupling of shared interests) helped to engender a degree of hybridity and interdependent learning between diverse actors. Therein, classical response actors were not usurped by the involved militaries, nor ostracised from the NERC and DERCs they established. Rather, the militaries helped to provide them an enabling environment in which to intervene, apply their technical expertise, and implement response activities (i.e., to practice their quotidian ritual interaction and thereby sustain their social organisation in an adapted way). The centres thus facilitated the accommodation of anomaly by its actors, rather than leading them to schism or exhaustion.

### Approaching the grassroots?

How hierarchy facilitated inclusivity, decentralisation, and robust coordination is now examined.

#### The virtuous cycle of inclusivity and robust hierarchical coordination

As previously described, as the number and diversity of involved actors increased in Sierra Leone’s Ebola response (as facilitated by the hierarchical NERC and DERCs’ inherent co-dependence and other conflict attenuating mechanisms), so too did the perceived need for more and better oversight of those actors. This followed from the perceived need to ensure that the various actors’ interventions were appropriately aligned, effectively and efficiently applied, and sufficiently accountable. The NERC and DERCs helped to resolve the perceived need they created therein, in that these centres’ hierarchical structuring cultivated a culture of discipline and accountability through the C2 structure itself, in and through which the Ebola response’s various components could more effectively and efficiently coalesce (though, while the militaries did not command or control within the NERC and DERCs, their presence did arguably have this effect on Ebola-affected populations. For example, RSLAF had the power to enforce or coerce compliance of public health measures including quarantines).

**Fig. 3:**
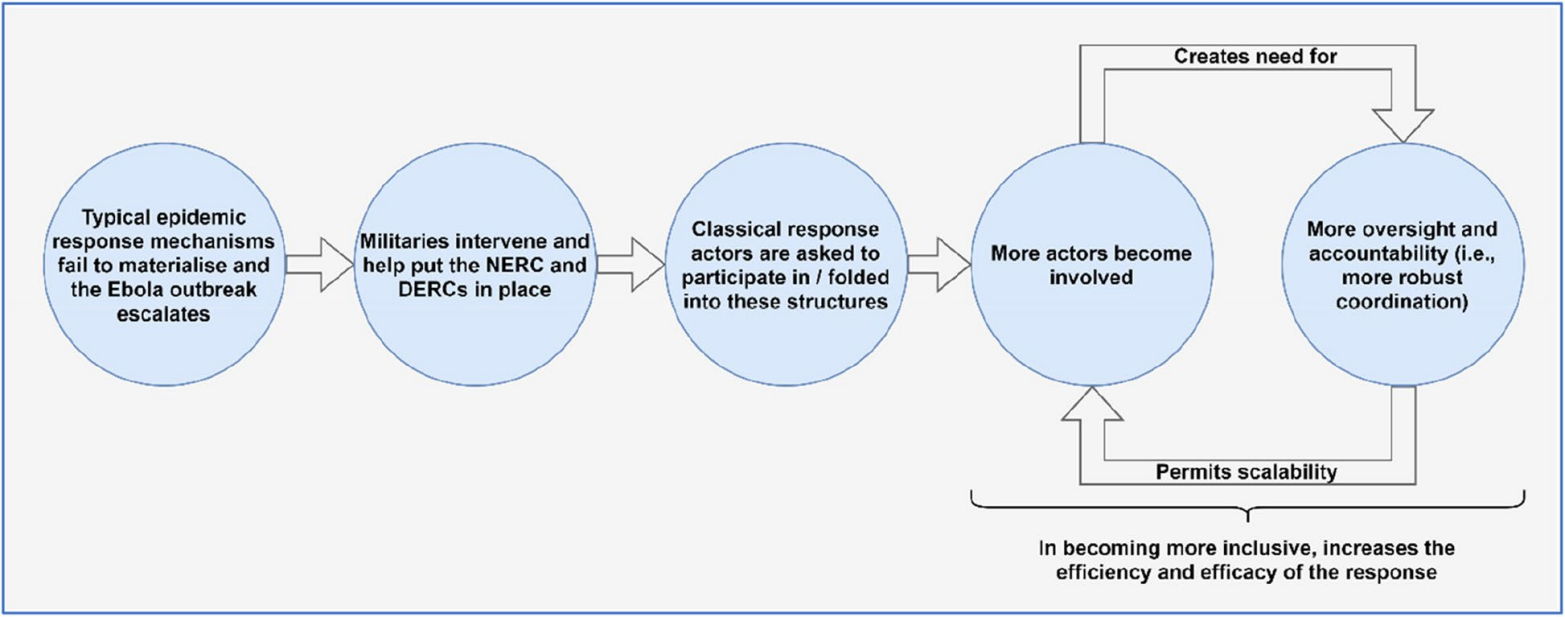
The virtuous cycle of inclusivity and robust hierarchical coordination (Source: author)

In turn, the discipline and accountability that was imparted through the NERC and DERCs (as well as the conflict that was attenuated between its diverse actors) amplified the response, as it permitted the further safe and effective delegation of response interventions to even more classical response actors. Through scaling it, this increased the efficiency and efficacy of the Ebola response (Fig. 3).

In other words, oversight and accountability created in the NERC and DERCs helped to facilitate the arrival and inclusion of classical response actors (which were comfortably able to apply their interventions due to the NERC and DERCs’ conflict attenuating mechanisms). Once their activities were aligned and accountable—which required the co-constitutive strengthening of coordination—there was the capacity to include yet more classical response actors. Essentially, this follows from the notion that co-dependence—when purposefully developed and encouraged—can be understood as a kind of inclusivity. One GoSL civilian respondent (who was an Ebola response leader) alluded to this, saying:

*I would disagree with anyone who suggests that the* [NERC] *and the military ended Ebola. No! We were not Ebola experts. It was the doctors from the* [MoHS] *who were the ones that led on the Ebola fighting… We thought that the experts—the epidemiologists, the medical doctors, the infectious disease doctors, the social mobilisers, the people who knew what to do to stop Ebola—they were the brain. We, at the* [NERC]*, we were the muscles* (GoSL-C-N-17).

In other words, the NERC and DERC “*muscles*” meant the militaries could (generally) limit their role to ensuring oversight and accountability, while classical response actors could intervene and then perform a diverse array of ongoing activities within the bounds of their respective pillars by applying their “*brain*” (GoSL-C-N-17). Taken together and put simply, military and classical response actor skillsets were—in the words of a British Armed Forces respondent—felt by most respondents to “*combine for the greater good*” (HMG-M-D-4). That is to say, where the presence of each actor encouraged, facilitated, and strengthened the presence of the other.

#### Marginal(ising) inclusivity of more local actors

While the NERC and DERCs were therefore inclusive of some actors, these structures were exclusive of others, because the intervention of the centres’ military and classical response actors had a marginalising effect on some local communities affected by the epidemic. However, in Sierra Leone’s Ebola response (as discussed below), a degree of localisation and empowerment of more local actors and groups did occur. Indeed, hierarchical and military decision makers proactively incorporated and supported Paramount Chiefs, District Security Committees (DISECs), (sub-)chiefdom task forces, and thousands of Sierra Leonean ERWs to become participants in the Ebola response. This, in turn, supports the proposition that conflict attenuating hierarchical structuring can permit a degree of decentralisation to and inclusion of more local groups in public health emergency responses, thereby mitigating the trend of marginalisation of community-based actors that is otherwise implicit.

From early on in the outbreak, Sierra Leone’s network of chiefs were co-opted into the formal Ebola response to help ensure local populations complied with Ebola-related restrictions. This inclusion did sometimes present a challenge to these traditional leaders. For example, some were frustrated by feeling made to participate in the Ebola response (especially as enforcers of public health measures), as this resulted in some animosity amongst their constituents. Others were frustrated because they felt insufficiently supported financially, despite being asked to perform activities that sometimes had cost implications. Other scholars, meanwhile, have examined how the response itself re-arranged (and in some ways challenged) local conceptions of public authority, including the chieftaincy structure [12].

Nevertheless, despite being relatively limited, processes of local inclusion did occur through the involvement of the Paramount Chiefs (at least in the areas of north-western Sierra Leone where data was collected. Other scholars have noted that findings might not be generalisable elsewhere in the country, especially in the east) [13]. A GoSL civilian respondent (and senior decision maker) recalled how

*…we got to a point where… military aides* [were provided] *to Paramount Chiefs. We led the Paramount Chiefs to believe that the military aide was there to protect them, and to some extent, that was their job. But… their real job there was to help the chiefs be more efficient.* [So] *when* [the chief] *is calling meetings of* [their] *elders, when* [they are] *deciding what to do, when they are discussing* [something]*, to inject that military officer into* [the discussion]… *that had an effect on how the chiefs organised themselves,* [and] *how the villages organised* [them]*selves. Because you just need that one planner, that one logistician, and then, pretty much, people can help themselves* (GoSL-C-N-17).

In other words, NERC leadership deployed military personnel to support the various Paramount Chiefs, and the presence of the soldiers’ hierarchical thought style was felt (at least to this senior decision maker) to acculturate not only an ethos of efficiency amongst the chiefs, but also to empower a degree of self-reliance for them and their communities.

The perceived need for this intervention is discerned by Douglasian Theory (in reference to hybridity), which argues that

…if we are to live together in ways that will enable us to channel our conflicts into more civilised and restrained practices, we need to dance our common time to each other’s rhythms as well as our own [5].

Accordingly, the respondent quoted above (GoSL-C-N-17) believed it was necessary to provide resources and structure to those with slower rhythms, so as to make them more efficient in response to the crisis at hand. In doing so in this instance, the complementarity of local inclusion and efficiency became not only possible but desirable, even from the perspective of one of the Ebola response’s most senior decision makers (GoSL-C-N-17).

Paramount Chiefs largely agreed that the presence of military ERWs was generally empowering in nature, including for the psychological effect it had. For example, one felt the presence of the military “*motivated every individual to take the whole event as a very serious one*” (PC-C-C-1); another that “*interaction* [with military personnel]… *aided moral*e,… *helped to create a more positive mindset,…* [and] *increase*[d]*… confidence* (PC-C-C-6); and another that the military personnel “*were able to console us, talk to us, and persuade us to have faith within ourselves…”,* concluding that *“…seeing them… summon*[ed] *up courage*” (PC-C-C-4). In other words, the intervention and subsequent presence of military personnel alongside Paramount Chiefs was felt by most to impart a degree of confidence. Notably, several Paramount Chiefs suggested that—due to Sierra Leone’s history as a former colony of Britain, the British colonial administration’s role in reinforcing the country’s chieftaincy structures (from which Paramount Chiefs derive their authority), and the role the British Armed Forces played in ending the Sierra Leone Civil War—they were not only grateful for but actually expected the militarised support from HMG that they received (PC-C-C-1; PC-C-C-2; PC-C-C-6). One Paramount Chief, for example, stated that

…*whenever we cry,* [the British] *should cry too… Because of the operation they carried… in the war…, whenever we have a situation or crisis, we expect the British military to come… We see that in all the crises Sierra Leone has ever had… It is clear in the minds of the people that whenever we have a crisis, the British… military has to come before we are able to see headway…* [and] *Ebola, again, is the same thing… When the British military came in… the people have that belief that… the situation was going to be over… Ask any Sierra Leonean, and they will tell you this* (PC-C-C-6).

Therein, to this Paramount Chief, the intervention of the British Armed Forces specifically (i.e., as distinct from RSLAF) had a significant psychological component, in that it was felt to evidence Britain’s empathy, and gave them confidence that the crisis would be inevitably resolved.

These feelings of assurance were greatly aided through the resources that military personnel were able to facilitate for Paramount Chiefs. One, for example, remarked that in the (pre-DERC) DMO-led response, they

…*hardly* [got] *some of the things…* [they]*… ask*[ed for] *to strengthen the community in the push for this Ebola* [eradication effort]*… But when* [they] *reach*[ed] *any lieutenant or captain in the military* [who was situated in the DERC]*,* [the military personnel] *say, ‘Why not? Why don’t you get this?’. Everything* [was] *available* (PC-C-C-1).

That is, the DERC’s military personnel were able to secure the delivery of tangible resources for Paramount Chiefs in a way that the pre-DERC’s civilian personnel were not, and to this Paramount Chief, did so proactively and with encouragement. This was felt, in the words of another Paramount Chief, to “*guarantee our effort to go and do* [Ebola response activities]” (PC-C-C-6). It is important to reiterate that the militarisation of the Ebola response corresponds with the time when significantly more resources were made available to Sierra Leone (i.e., that HMG’s intervention was both civilian and military in nature, and the availability of resources at a local level was not necessarily due to the latter). Nevertheless, the DERCs’ military personnel were the focal point through which these Paramount Chiefs requested and secured the resources they needed in a way that they previously could not. This helps to explain why Paramount Chiefs generally associated the availability of Ebola response resources with military ERWs, rather than their civilian counterparts.

Notably, as an important component of Paramount Chiefs’ positive interaction with military personnel—and echoing the organisational hybridity previously examined—several commented on how the response’s civil-military makeup softened the militaries’ approach and made the militaries’ role more tolerable. One Paramount Chief, for example, remarked that, prior to the outbreak,

…*the military was just* [perceived as a] *sort of threat. But with*… [civilian responders] *mixing with our brothers in the military, you know, talking to our people,* [and] *visiting areas,…* [then people] *felt very comfortable… Having a mixture of the military personnel, the foreign*[ers]*, and the indigenous… made everything okay* (PC-C-C-1).

In other words, to this respondent, there was the possibility that the presence of military actors could have presented a problem for them and their constituents (i.e., they may have been perceived as threatening by the population). However, this problem was felt to be mitigated by the incorporation and joint effort of these actors alongside civilian ERWs, which to them, demonstrated the peaceful role the military actors were performing in the Ebola response. Another Paramount Chief echoed and elaborated on this notion when they remarked how surprised they were by how amicable and obliging the DERC’s military personnel were:

*The idea… before* [the Ebola outbreak]*…* [was that] *military* [personnel are] *somebody that cannot laugh and cannot talk to anybody…* [But] *the first time I met these military personnel* [in the DERC]*, you* [could] *not know* [that] *these people* [were] *military personnel, because the way they* [did] *things… The way they talked to people, and the way they responded to issues… The military were so kind and peaceful, and they did not even behave like the military. They were so soft… I cannot over-emphasise their kindness, their behaviour, and the human character* [they exemplified] *towards mankind*… [They] *made us understand that the military* [personnel were] *just normal human beings, and the only difference we* [civilians] *have* [compared with them] *is the discipline* [they manifest]*… Maybe…* [this is] *because of the partnership* [the militaries had]*… working in the same office* [as civilians]*…* (PC-C-C-6).

To this respondent, therefore, the military personnel involved in the Ebola response were perceived to be hospitable when compared with past experiences or prior assumptions (something which they theorised was due to fact that military personnel were working alongside civilians on a day-to-day basis, i.e., that their approach was moderated through organisational hybridity). One Paramount Chief even remarked that the DERC’s military personnel “*listen*[ed] *to you more than even our own people*” (PC-C-C-1).

Therein, to several Paramount Chiefs, the civil-military nature of the Ebola response served to humanise its military actors. It also, according to one Paramount Chief, served to humanise Ebola-affected communities:

*To me, I will always say that, if the… military had not intervened in the fight* [against] *Ebola, nobody would believe that* [an] *Ebola-affected person* [was] *not a criminal, is not a condemned person, and is* [actually] *just like any other person. Because* [the military personnel] *would come and interact* [with the Ebola-affected person]*…* [with] *limited barriers* (PC-C-C-6).

That is, to this Paramount Chief, the response’s military personnel (in this case, those maintaining quarantine cordons) interacted with quarantined individuals. Provided the significant stigma and fear that was often associated with Ebola-affected families, this was felt to de-vilify them. While specific descriptions of these dynamics were limited (due to the fact that research primarily focused on documenting intra-DERC CMRel rather than field activities), other Paramount Chiefs and a minority of classical response actors also recalled instances when military personnel went beyond their mandate to not only secure but proactively support quarantined households, such as by fetching water, providing psychosocial support, and tending the affected family’s farm (and therein, protecting their livelihood while they were in mandatory isolation).

Overall, Paramount Chiefs were notably supportive of the militaries’ presence in the Ebola response. Most felt it imparted confidence (that some expected was forthcoming due to the UK’s historical relationship with Sierra Leone); facilitated and secured tangible resources; and was gentle and moderated in nature—perhaps due to civil-military mixing—in a way that was sometimes seen to extend to vulnerable Ebola-affected families. One Paramount Chief summed up working with the Ebola response’s military personnel accordingly:

*They had smiling faces, they were friendly… They were able to give hope to people… You see them always active, and want to do things* [on] *time… to see that things happened and the problem*[s were] *solved… Even if you had any concern and you* [went] *to the* [DERC]*, you would be perfectly received by them with a smiling face ready to listen to you, and ready to solve your problem. Immediately,… you would see their commitment,…* [and] *they would communicate to the responsible* [person] *and say ‘This is what the Paramount Chief… wants’. That is how I believe they help*[ed] *the people to come out of the* [Ebola outbreak]… *So, if* [one is to] *rate the participation of any participants in the Ebola response, the military will be the first of the people or groups that help*[ed] *to eradicate Ebola in Sierra Leone* (PC-C-C-6).

Ultimately, all Paramount Chiefs that were interviewed (*n* = 6) were net-positive about the intervention of both RSLAF and the British Armed Forces, and stated they would want the same (or a greater) military role in response to a hypothetical future crisis.

Importantly and as captured in the data, processes of decentralisation and more localised inclusion in Sierra Leone’s Ebola response amounted to more than the participation of the country’s network of Paramount Chiefs.

Each district of Sierra Leone has a DISEC, a network of structures that was in place prior to the Ebola outbreak (these were established as part of the post-civil war and HMG-supported security sector reform (SSR) that included the military-military officer training programme (ISAT). These structures formalised the role of Paramount Chiefs in Sierra Leone’s security apparatus (GoSL-M-D-2; GoSL-M-D-10; GoSL-M-N-6)) [14]. According to an RSLAF respondent responsible for helping to oversee these structures, when the Ebola outbreak began, DISECs were

…[already] there… They have the power and mandate to invite anybody that has to do with something of the issue that is being addressed… [For example] women’s organisations… [and] international organisations on the ground, they are automatically part of the DISEC process (GoSL-M-N-14)*.*

Therein (and as corroborated by a Paramount Chief), DISECs are comprised of not just local public authorities and chiefs, but also local civil society organisations (CSOs), youth councils, women’s leaders, local human rights monitors, et cetera (PC-C-C-2).

At first, these structures were poorly integrated into the Ebola response—in the words of one Paramount Chief and DISEC member, controlling Ebola “*from Freetown, no, it did not work until we had the* [DERCs]” (the DERCs took up to 2 months longer to establish than the NERC, but once in place, formally involved the DISECs and other local structures) (PC-C-C-2). *“Then it started working*”, they continued (PC-C-C-2). Once online, the DERCs more purposefully involved the DISEC network, as well as the growing number of Ebola response community task forces. Weekly meetings between Paramount Chiefs and the DERCs were also put in place, in which Paramount Chiefs were made active participants in district-wide Ebola response decision making (PC-C-C-3) (note—as referenced above and also as discussed in this article’s limitations section—the experience of Paramount Chiefs in north-western Sierra Leone is not necessarily generalisable to the experiences of local authorities elsewhere in the country).

These community task forces—the formation and operation of which were funded through the Ebola response—became well established and highly structured: bigger towns were broken down into smaller sections; areas with higher rates of Ebola received extra attention; and sectional sub-task forces were established, such as those which were solely comprised of youth groups (PC-C-C-2). One Paramount Chief described the process of the task forces’ inception and utility, the way in which it was hierarchically structured, and the relative diversity of its localised participants:

*As Paramount Chiefs, we are… always with our people… I was part of the first task force that was formed in the* [Government] *hospital by the hospital staff… We had the first meeting before the disease came into the district. So, we were well informed and well prepared beforehand… And also later on,* [GoSL] *involved the Paramount Chiefs to take part by bringing out the Ebola bylaws* [which were] *designed by the National Council on Paramount Chiefs… The Ministry of Local Government came out with a document that we have to set up a chiefdom task force and a town task force and a village task force… So, I formed the chiefdom task force, wherein I have all the section chiefs…* [as well as] *a women’s leader, the pastor, the imam from the mosque, the youth leader, two members from the medical field, one herbalist, a journalist, the teachers, the motorbike rider’s association,* [and] *the driver’s union. Because these people are very important in the fight against Ebola* (PC-C-C-2).

In other words, this chiefdom task force (which reported to the DERC) was itself comprised of town task forces and village task forces, which were themselves comprised of a large number of diverse local actors. These community task forces monitored movement and quarantined homes; set up night-time checkpoints (for which, in this instance, they were given a tea and head torches by the DERC); and supported contact tracing efforts (PC-C-C-2). At times, they went so far as to monitor the DERCs’ contract tracers themselves to make sure they were performing their jobs appropriately (PC-C-C-2). The task forces also ensured a crucial degree of ground truth and local knowledge to classical response actors in the DERC, for example, by arguing for the ability (and providing the necessary oversight) for communities to bury their dead in a safe and dignified way (GoSL-C-N-24). The community task forces were, in short, profoundly important community-owned and community-led Ebola response organisations.

Importantly, the research sites’ community task forces were not in parallel to the formal Ebola response, but rather, were integrated with it. For example, the NERC- and DERC-organised Kambia [District] Community Action Plan (KCAP) leant on both Kambia’s DISEC its various community task forces to access and involve communities at the most local levels, including women’s groups, youth groups, and—in the words of an involved Paramount Chief—“*just about everyone*” else (PC-C-C-6) [15]. Recalled a GoSL civilian respondent (and senior decision maker):

That was part of the decentralisation thing. You don’t have to be a rocket scientist… To involve the traditional leaders,… the Paramount Chiefs and their section chiefs, and community people, village chiefs… They all played a very critical role… It was a true team effort (GoSL-C-N-24)*.*

At least to this respondent, therefore, decentralising the Ebola response in a way that formally included community initiatives and structures in this way was fundamental to the overall success of containing Ebola and, ultimately, ending the epidemic.

The degree of localisation in the Ebola response is perhaps most clearly evidenced by the sheer scale of the Ebola response’s workforce, as facilitated by its hierarchical actors. For example, upon their arrival in late 2014, medics from the British Armed Forces and RSLAF established two sites in Freetown to train Sierra Leonean ERWs in biohazard protection. On completing this training, individuals could then safely participate in the variety of Ebola response roles requiring the use of Personal Protective Equipment (PPE), which ranged from ETC hygienists, to ambulance drivers, to decontamination and burial team workers, et cetera. More than 4200 individuals—a significant number for a country with so few medically trained individuals—were trained in these military-led centres, each of whom was given the instruction they needed to become safely participant in the response. This not only made the response more ethical for the degree of localisation it facilitated, but also more efficient and effective as it accelerated subsequent scale-up.

It is important to emphasise that the Ebola response did not perfect this localisation. Indeed, the legacy of the response is one of marginalisation as much as it was one of inclusivity, including from the perspective of some Paramount Chiefs. For example, despite the inclusion of Paramount Chiefs, DISECs, and community task forces, one Paramount Chief said communication between military ERWs and local actors could have been much improved (PC-C-C-5), and another spoke about the ways that inclusion of local actors could have been more robust (P-C-C-2). The latter, therefore,

*…recommended strongly that the traditional leaders or rulers have to be incorporated in disaster management, because disasters hit our people. It doesn’t hit the higher office… When you bring in people like the army, the police, and the medical experts from overseas* [such as the] *WHO* [or] *UNICEF… They will be working with the local people, and there is always that gap … So, we have to come in to narrow that gap…* [in] *ethnicity, language…, tradition, and culture…* [by] *involv*[ing] *people on the ground* (PC-C-C-2).

This respondent argues, in other words, that local people will always be primarily affected by an emergency, and must therefore be proactively integrated within the response in a way that better ensures local dynamics are respected, knowledge utilised, and expertise empowered. However, their recommendation to involve and empower more local people in the response to future crises is, to an extent, a lesson that was learned: post-Ebola, this same Paramount Chief was sent abroad with a cluster of other Sierra Leoneans to be trained in disaster management (PC-C-C-2). In other words, community-level actors were recognised for their import in the Ebola response, and subsequent efforts were made to further empower them. In the words of another Paramount Chief,

*…the Ebola response has proven that with the empowerment and the development of the chiefdom administration,* [GoSL] *can achieve its objectives in terms of development, in terms of disease control, in terms of education, and in terms of everything else. Because in the chiefdom we have a structure, and this structure cuts across to the last village (PC-C-C-6).*

In short, however limited it may have been, the decentralisation and localisation of Sierra Leone’s Ebola response to (some) empowered sub-district local actors was made possible through the strengthening of hierarchical coordination, as the need for robust coordination became more pronounced the further that decentralisation occurred (and in turn, the further that activities were scaled). However, decentralisation did not mean less hierarchy, but more: as the NERC oversaw and supported the DERCs, so the DERCs oversaw and supported the more local Paramount Chiefs, DISECs, and community task forces. After all, according to Douglasian Theory,

…in hierarchy… there is a multiplicity of levels, quite contrary to the common misunderstanding of hierarchy as comprising only high-status commanders and the commanded who lack status [5].

Accordingly, and as stated by a British Armed Forces respondent, the hierarchy presented by the NERC and DERCs was not about removing the function of less hierarchical and more local actors, but—at least in the areas where data was collected—supporting them in a way that “*was just about speeding things up*” (HMG-M-N-2). In conclusion, an (I)NGO respondent argued that “…*it was the DERCs and it was the NERC that got rid of Ebola. That is the truth* (NGO-C-N-7).

## Discussion

The British and Sierra Leonean militaries played a central role in responding to the 2013–2016 West Africa Ebola Epidemic in Sierra Leone. For the extent of CMRel that were manifested, the intervention was unique, and to many, controversial. The aim of this article—through the application of Douglasian Theory to 110 semi-structured qualitative interviews with civilian and military ERWs—is to not only better understand these dilemmas, but to derive lessons as to how negative effects might be mitigated or interrupted.

Pertinent concerns in the literature coalesce around three key themes and debates.

The first key theme and debate relates to scepticism regarding the role of militaries in humanitarian crisis and public health emergency responses, especially the various harms that militarised interventions arguably risk. For example, some scholars argue that by intervening in such contexts militarily, it is very difficult—if not impossible—to consistently adhere to the Humanitarian Principles of independence, impartiality, neutrality, and humanity [16–18]. This is evident, for example, when classical response actors rely on military assets (thus losing their ability to claim ‘independence’) [19–23]; and also when military actors supporting humanitarian or public health interventions commit acts of violence against crisis-affected populations (thus nullifying ‘humanity’) [24–27]. Many scholars argue that this, in turn, puts both classical response actors at risk of harm, and also limits their ability to provide life-saving assistance in response to the crisis at hand [17, 18, 28]. Other scholars have argued that this kind of militarised intervention also contributes to the problematic ‘securitisation’ and ‘militarisation’ of humanitarianism and global health [29–33].

The second key theme and debate relates to arguments that—irrespective of these risks—productive and effective civil-military cooperation is untenable due to key organisational differences between classical response and military actors [34–36]. In particular, scholars highlight the different approaches to hierarchy that the actors manifest. That is, classical response actors are generally characterised in the literature as horizontally organised and bottom-up organisations, which are democratic and consensus-based by nature [37–39]. Militaries, on the other hand, are characterised as wholly hierarchical and top-down organisations, with dictatorial decisions being implemented including through the use of coercion [35, 40, 41]. Scholars argue this difference results in a significant lack of trust, and an overall challenge—if not an impasse—to effective and productive civil-military cooperation in response to humanitarian crises and public health emergencies [16, 34–36, 40, 42, 43].

The third key theme and debate relates to the assertion that exogenous interventions impede capacity building amongst local institutions and actors (in turn limiting their resilience to future crises) [44–46]. For example, some scholars argue that if and when classical response and military actors appropriate the coordination and delivery of key health services, local institutions with relevant mandates—such as a health ministry and the wider health system—are unable to practice their *raison d’être* [47–50]. This may limit local staff’s ability to learn from the crisis, and may also mean response funds are primarily conveyed to classical response actors (that may or may not remain in situ post-crisis) [48, 51–53], rather than to an under-resourced health system in need of financial assistance [2, 54, 55]. Both effects may decrease local institutions’ resilience to future crises [45, 56, 57]. Relatedly, scholars also argue that exogenous interventions ignore more local actors (especially the least politically empowered ones), which can: exacerbate their marginalisation and existing inequities [3, 58, 59]; mean responses are less adaptive and sensitive to important local context(s) [7, 9, 60]; and mean responses do not take advantage of endogenous capacity and other strengths that would otherwise represent essential contributions to the response at hand [4, 6, 61].

For all these reasons, in the data, the militaries’ intervention in Sierra Leone initially frustrated classical response actors (as briefly summarised here, this is reflected on at greater length in other articles being published soon). Popularly conceptualised as highly distinct from militaries, they felt their professional territory was encroached upon. However, over time, this civil-military challenge was largely overcome and replaced by civil-military cooperation and collaboration [62]. In large part, this was due to surprising similarities in the hierarchical underpinnings of these groups’ cultures of interventionism and forms of organisation, institutional power, and thought style. This is highly disruptive of the second key theme in the literature. While these similarities may have resulted in straightforward CMRel, it also implicates classical response actors in the vicious cycle of life-saving assistance and structural harm that can play out during top-down public health emergency responses. One of these harms in Sierra Leone was the insufficient inclusion of crisis-affected local populations therein (as also examined by other scholars) [3, 7, 8, 63]. Technical and operational efficiency was privileged over processes of inclusion which were perceived to be cumbersome, slow, and ultimately counterproductive to the goal of saving lives—in a sense, recognition that the ‘principle of do no harm’ cannot fully apply if life-saving interventions are performed.

To do so, it was first necessary to understand the ways in which the hierarchical NERC and DERCs were purposefully designed with the support of military personnel from both the UK and Sierra Leone to accommodate a wide array of different groups, as it was understood that the inclusion of and collaboration between diverse actors would improve day-to-day decision making. This was felt to require robust oversight and accountability, as well as (relatedly) the standardisation of activities guided by best practice, both of which were also realised through the NERC and DERCs.

However, in the data examined in this case, this oversight and accountability was enabling rather than disrupting, as the centres—in and through which classical response actors and militaries interacted—were conflict attenuating spaces. Their inherent hierarchy permitted and employed the use of rule-bound niches, neutral zones, co-dependence, and hybridity to help ensure that the diverse group of actors within could cooperate without incessant and disruptive conflict. This, in turn, helped to ensure that each group could continue to practice their quotidian ritual interaction.

Therein, while the NERC and DERCs might have nominally embodied a C2 *modus operandi*, its hierarchical actors tended not to rely on confrontation or coercion. Rather, the management and coordination style of the Command Team was much closer to “coaching”, to borrow briefly from Campbell’s popular management terminology, wherein a desired approach was acculturated amongst others rather than mandated [64, 65]. Clarke and Campbell see this as decision making best practice in humanitarian contexts, wherein effective decision making occurs through the decentralisation of operations, provided decentralised authorities are provided proper guidance and SOPs (which are themselves a kind of ritual ordering, to use Douglasian terms) [66]. Interestingly, it is also not entirely dissimilar from some military doctrine, including the British Armed Forces’ mission command structure, wherein responsibility for daily decision making is devolved to lower level operatives who

…are told what to achieve and why, but are then left to decide how to achieve it. Subordinates are encouraged to use their judgement, initiative, and intelligence in pursuit of the commander’s goal [67].

Therein, instead of hierarchy being used to give top-down orders, it is used as a structure for the dissemination of intent and resources—decision making itself is delegated (i.e., it is allowed to occur in a more decentralised way). This coupling of coaching or guidance and localisation that is achieved through hierarchy is crucial, because anthropology and sociology have repeatedly found that top-down confrontation and coercion leads to greater resentment, revolt, and, eventually, to “more ferocious enclaving” [5].

**Fig. 4:**
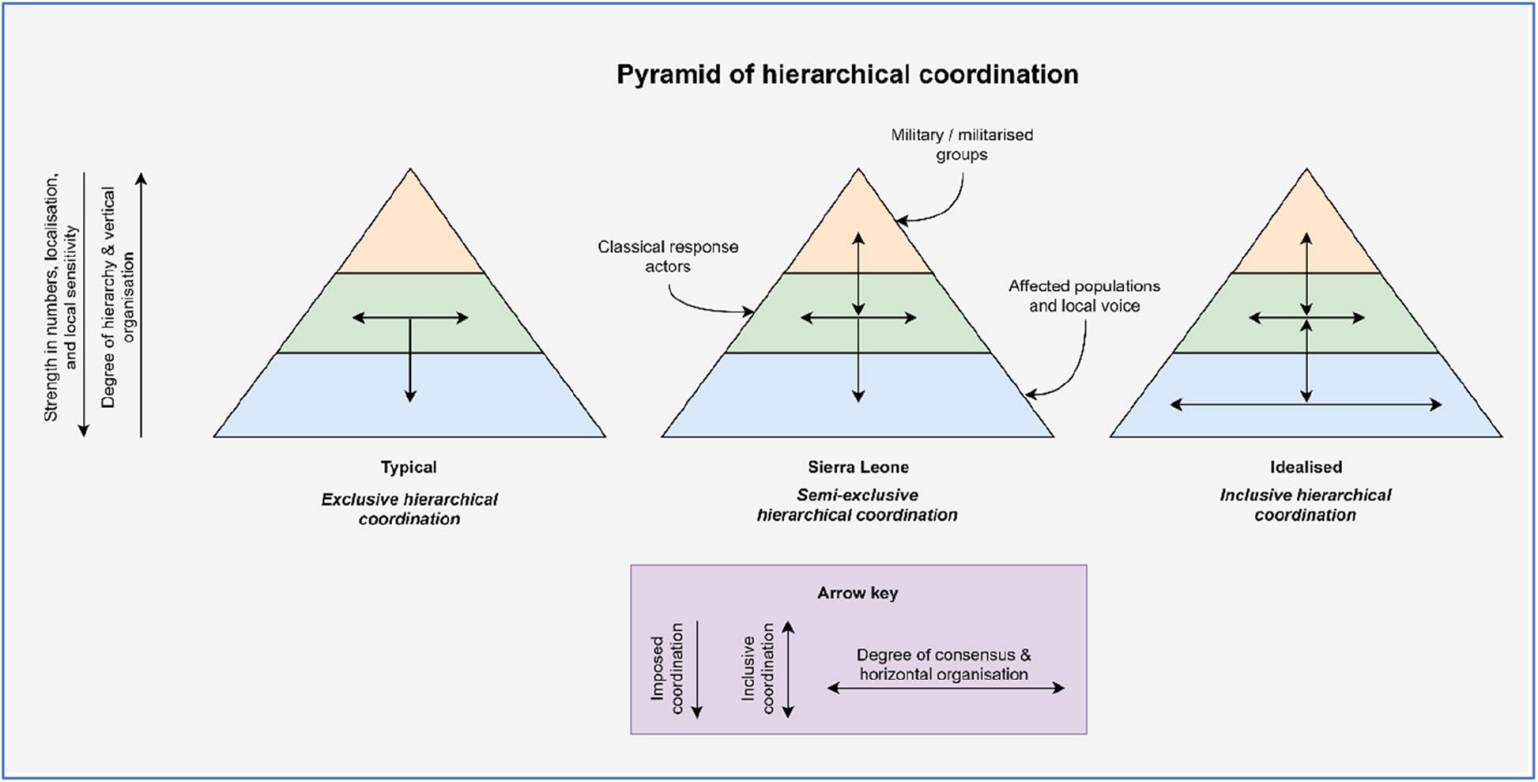
Typical exclusive, Sierra Leonean semi-exclusive, and idealised inclusive hierarchical coordination (Source: author)

This ‘semi-exclusive hierarchical coordination’—in which hierarchical classical response actors were made to coordinate with even more hierarchically organised militaries—did, to some extent, still impose Ebola response coordination on local populations in a top-down manner (Fig. 4). Indeed, the actors taking decisions to put the NERC and DERC structures in place were still hegemonic ones, and did so without the systematic input of many national health actors let alone local communities. Harm therefore resulted from this exclusion and marginalisation of local groups from full ownership of what was ultimately their public health crisis. In other words, what was seen did, in some ways, aligns with the third key theme and debate (that exogenous interventions impede capacity building amongst local institutions and actors).

However, when taking the perspective of militaries in this instance, the NERC and DERCs also evidence an important willingness of the hierarchical actors (albeit measured) to reach down and include less hierarchical groups in response to the Ebola crisis. Indeed, several of the most hierarchical actors involved in Sierra Leone’s Ebola response considered it both efficient and effective to accommodate and proactively support such groups (at least in the thesis’ areas of data collection). Doing so permitted a degree of decentralisation, localisation, and scalability. While this inclusion was limited, it was not nominal: hierarchical actors committed human and financial resources to these local actors, and proactively incorporated them into response structures.

Toning down a fully militarised C2 approach was key (as argued by other scholars, so was the militaries’ de-emphasis of security logics [68], which was perhaps made more straightforward for the fact that the British Armed Forces did not deploy combat troops to the Ebola response. Whether this was evident to affected populations is less clear). Equally important, though, was supporting a more disciplined and efficient approach amongst classical response and other local actors so as to meet in a kind of middle ground: through the NERC and DERCs, oversight and accountability was extended beyond what would otherwise have been possible, efficiency and discipline was acculturated, and the response grew to thousands of (primarily Sierra Leonean) workers at the country’s national, district, and sub-district levels. Once respectful relationships were established between these diverse actors, mutual learning was made possible. This, in turn, helped to ensure co-dependence was inherent in day-to-day activities. As it became clear to classical response actors and some local actors that their participation in the NERC and DERCs’ hierarchical scheme was made secure through this co-dependence, anomaly (presented by both the outbreak itself, and also the intervention of military actors responding to it) became accommodated.

Taken together—and perhaps counterintuitively—this leads to this article’s major contribution to the literature: a form of inclusivity in Sierra Leone’s Ebola response was made possible through the conflict attenuating hierarchical ordering of its coordination centres and the subsequent accommodation of and decentralisation to less hierarchically organised groups. The involved militaries, in other words, not only encouraged but contrived the routine and empowered inclusion of less hierarchically organised groups in the Ebola response (in this instance, primarily classical response actors), thereby permitting scalability in a virtuous cycle. In a sense, this is akin to Durkheim’s Division of Labour as not only the solution to inter-organisational conflict between differently ordered groups through mutual exchange, but also the most efficient mode of performing a multifaceted activity [5]. As previously described, it is also not entirely distinct from some military doctrine including the British Armed Forces military mission command, which through delegation, allows for the more efficient application of decision making in the pursuit of a stated objective. This finding is not only quite unique, but also somewhat disruptive of all three themes and debates in the literature: there was no widespread findings of harm to classical response actors or crisis-affected populations; organisational differences facilitated rather than impeded cooperation; and a form of localisation was made more straightforward, not less, through the militarised hierarchy.

Further advancement of this contribution can be theorised. If a military can purposefully structure itself and its operational environment to incorporate less hierarchical, more democratic, and more consensus-driven decision makers such as classical response actors and Paramount Chiefs in response to a high-speed and life-threatening crisis, classical response actors ought to be equally capable of extending this same inclusivity down the hierarchical spectrum. This should be possible even if one seeks to incorporate forms of social organisation that fall fully outside of the hierarchical type, such as marginalised and enclaved local groups (a phenomenon that Douglasian Theory has appropriately discerned) [5]. Doing so would help draw on the benefits of hierarchy reflected on by many respondents, while mitigating the risks of militarisation that are very present in this kind of intervention.

Therefore, while hierarchical coordination produced some harms in Sierra Leone, it perhaps did so for the ways it was imperfect and incomplete, particularly if and when the hierarchy “insists on grand unity” (such as the shared objective of responding to a large-scale public health emergency necessitating a whole-of-society response), and therein,

…allows scope for atonement, reintegration, and a more porous conception of the community open to individual or local group commitment and efforts to join [5].

In other words, it is not just conflict that is attenuated, but peace that is created: by joining hierarchical ordering with unity of purpose, a structure of interaction, cooperation, and collaboration can be created through which the boundaries between various groups are made more permeable and local groups can be empowered to become more centrally participant.

This, in turn, represents the possibility of a truly ‘inclusive hierarchical coordination’ in the response to future public health emergencies (Fig. 4)—one that draws on lessons from but certainly does not rely on nor require military intervention. To extend the system of hierarchy would have permitted further localisation through the extension of oversight and accountability, thereby improving the Ebola response through greater horizontal organisation at its lower levels, the localisation of its interventions, and strength in numbers. A truly representative democracy is ultimately a direct one, after all, at least for those who choose to be involved, and effective hierarchy provides a structure for not only top-down direction but also the provision of guidance and resources (as well as bottom-up advocacy). In short, if realised, this inclusive hierarchical coordination would retain its particular organisational strengths, but also become more ethical, efficient, and effective for the ways it would no longer impose coordination on crisis-affected populations in a top-down manner but rather structure itself to systematically include and empower them as genuine participants.

## Conclusions

Outbreaks with epidemic and pandemic potential have an increased interest in health security as part of an entrenchment of nationalist ideologies. This kind of protectionist approach can be characterised as defending domestic borders in situ. It can also, however, be characterised by international intervention, to mitigate the risk of another country’s crisis spilling over. This kind of securitised—and particularly militarised—response is controversial, including for how it characterises health crises as globalised security threats, and how this kind of intervention can serve to reinforce neo-colonial relationships and the chronic underfunding for national public health sectors.

This case study, however, shows there are opportunities to transform crisis response into something that can actually be beneficial—both for the efficacy of the response and containment of the public health threat, and for the safety, inclusivity and empowerment of affected and marginalised populations. To do so, it is necessary to recognise how hierarchy (on the one hand) and decentralisation and localisation (on the other) are neither opposing ideologies nor incompatible aspirations—that is, one does not preclude the other. Rather, applied together, these approaches can be co-dependent, interoperable, and greater than the sum of their collective parts, at least when organised in a conflict attenuating way.

This was evident in Sierra Leone’s militarised NERC and DERCs, which helped ensure the various Ebola response activities organised within their rule-bound niche pillars and neutral zone fora were effectively interlinked, coordinated, accountable, and adaptable. Further, while the militaries put a C2 structure in place within the NERC and DERCs, they neither commanded nor controlled, but instead cultivated and permeated discipline and efficiency amongst the centres’ various actors. Therein, the oversight and accountability structures that the militaries put in place permitted classical response actors to intervene more effectively and to scale more aggressively than might have been otherwise possible.

To most ERW respondents (both civilian and military), therefore, elements of hierarchical oversight and efficiency in the NERC and DERCs was of great importance to the successful, cooperative, and collaborative operation of the Ebola response. Further, these same structures also permitted a limited degree of localisation to more local actors, such as Paramount Chiefs and their chiefdom-based Ebola task forces. This localisation was admittedly limited (both in nature and also geography), but nevertheless had valuable ethical and operational consequences for the response. Crucially, it also evidences the plausible viability of further localisation during future public health emergency responses using a more thorough application of similar strategies—one that could be fully civilianised, mitigating very legitimate concerns surrounding militarisation, if these lessons were fully considered and actioned by relevant health authorities.

In short, to realise ethical, efficient, and effective public health emergency responses—ones that systematically include local actors, while also ensuring that the resources required to respond are applied at scale, are aligned, and accountable—hierarchy, decentralisation, and localisation should go hand-in-hand.

## Limitations

This study has various limitations, each of which is mitigated by the large number of interviews and relative diversity of respondents (and relatedly, efforts to reach saturation); the confidentiality of participation and anonymisation of statements provided; and the routinisation of reflexive practices throughout the research process.

In-country site selection was limited to the Western Area Urban (i.e., Freetown), Port Loko, and Kambia districts of Sierra Leone. These districts are among the more politically privileged, and were predominantly affected in the second half of the epidemic after many lessons had been learned. Therefore, the perception of the response is plausibly more positive than what might be documented elsewhere. Relatedly, while Western Area Urban, Port Loko, and Kambia were the last substantively affected districts of Sierra Leone, data was collected in 2017 and 2018 (i.e., 2–3 years after the Ebola epidemic had concluded). The temporal gap between epidemic and data collection might have introduced recall bias among the respondents, potentially affecting the accuracy of their reflections on events and collaborations. Memory decay, shifting perceptions, and post-event developments might influence their accounts. For example, immediate tensions that were present during the outbreak may have waned in respondents’ memory (conversely, this delay may also have facilitated useful reflection and/or an ability to reflect more openly on these tensions than might have been possible during the outbreak itself). As with others, this potential limitation is somewhat mitigated by the comprehensive sample size (*n* = 110) employed in the study, which allows for a more diverse and robust range of experiences and perspectives to be captured, enhancing the generalisability of the findings.

Respondent selection presents other possible limitations. Female respondents are significantly under-represented (though this reflects the gendered skew of the response itself). Governmental respondents—perhaps especially military ones—may have been guarded in their criticism of their militaries, public institutions, or public officials. Paramount Chiefs were spoken to, in part, as representatives of Ebola-affected communities, but their positionality as public authorities in this regard is complex, fluid, and contested [4, 12]. Local populations responded in significant informal ways to the Ebola outbreak, but the documentation of these perspectives or interventions was not systematically collected or examined in this study. Finally, many respondents were known personally to STB. This may have influenced subject selection (indeed, as described in the methods, personal connections were proactively used to identify initial respondents, after which a snowballing technique was used). However, due to mitigating measures taken, any selection bias is thought to be small in effect—indeed, the number of respondents that were interviewed actually represents an overall majority of NERC and DERC personnel, and there was a considerable cross-section of organisational affiliations represented. Ultimately, while a significant number of respondents were known to STB (*n* = 38), a much larger number (*n* = 72) were not.

## Data Availability

Data (i.e., interview transcripts) are confidential.

## Abbreviations

C2: Command and Control
CMRel: Civil-Military Relationships
CSO: Civil Society Organisation
DC: District Coordinator
DERC: District Ebola Response Centre
DHMT: District Health Management Team
DISEC: District Security Committee
DMO: District Medical Officer
EOC: Ebola Operation Centre
ERW: Ebola Response Worker
FOB: Forward Operating Base
GoSL: Government of Sierra Leone
HMG: Her Majesty’s Government
ID: Unique identifier
(I)NGO: (International) Non-Governmental Organisation
ISAT: International Security Advisory Team
KCAP: Kambia Community Action Plan
LSHTM: London School of Hygiene & Tropical Medicine
MoHS: Sierra Leone Ministry of Health and Sanitation
MSF: Médecins Sans Frontières
NERC: National Ebola Response Centre
PPE: Personal Protective Equipment
RSLAF: Republic of Sierra Leone Armed Forces
SSR: Security Sector Reform
STB: Lead author and researcher
SU: Stabilisation Unit
UK: United Kingdom
UN: United Nations
USG: United States Government
WHO: World Health Organisation
